# Supplementary material for: PRDM16 co-operates with LHX2 to shape the human brain
Source: Oxf Open Neurosci. 2024 Jan 24;3:kvae001. doi: 10.1093/oons/kvae001 (PMC10914218; doi:10.1093/oons/kvae001)
Supplement: Web_Material_kvae001 [file web_material_kvae001.zip › Supplementary figures_revised.pdf]

SUPPLEMENTARY FIGURES

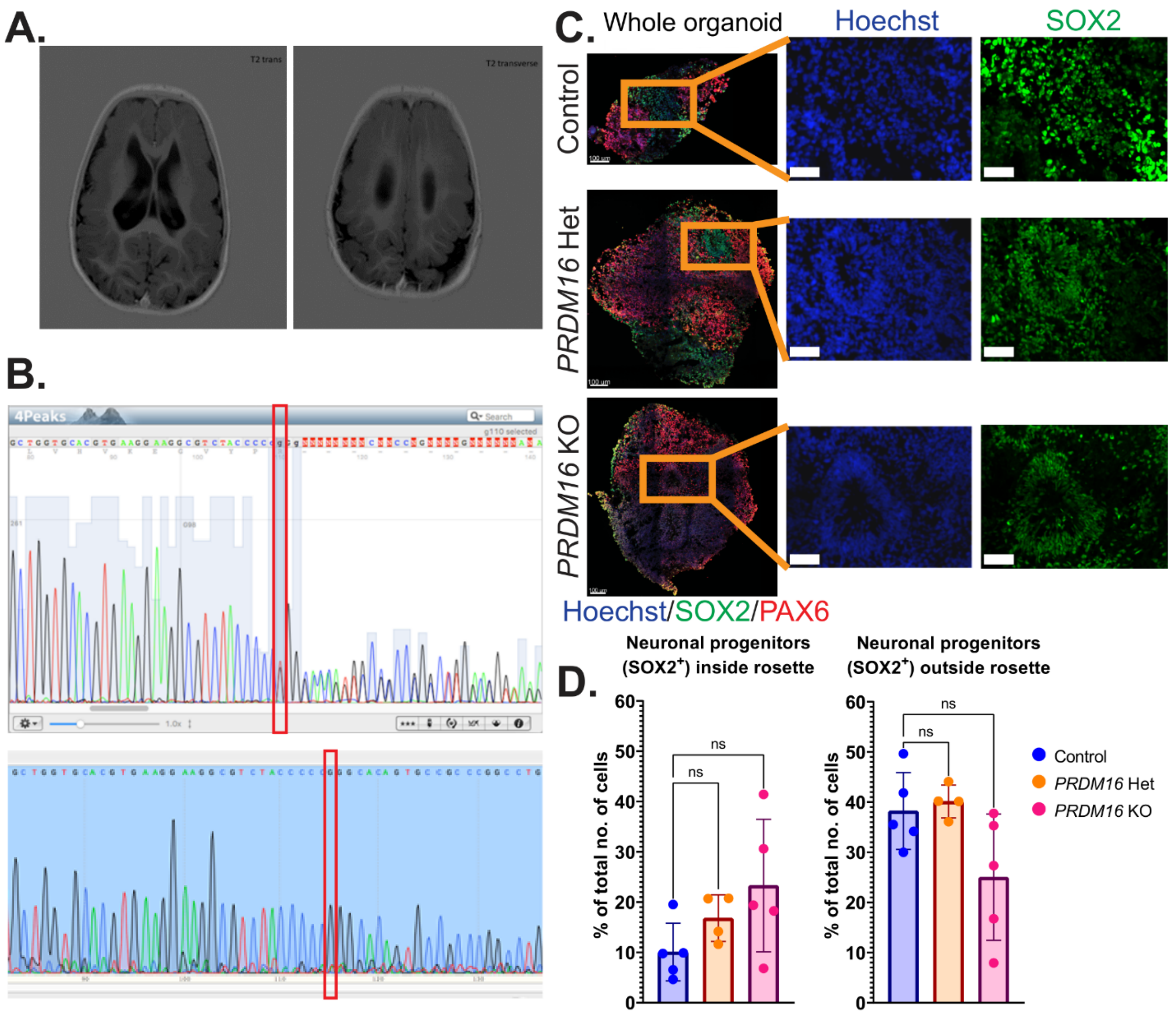

Supplementary Fig. 1

A. Additional MRI scans from the patient. **B.** Chromatograms of Sanger sequencing showing on the top the mutation in the patient's iPSCs (heterozygous), and on the bottom one of the homozygous hESCs PRDM16 mutated lines. **C.** Immunohistochemistry on 12 µm cryosections of day 32 control and *PRDM16* mutant cortical organoids showing the cortical progenitor markers – SOX2 and PAX6 (imaged using 25X objective lens, scale bar represents 50 µm in insets) **D.** Percentage of total number of cells that are $SOX2^{+}$ neuronal progenitors inside and outside the rosettes, in day 32 control and PRDM16 mutant cortical organoids (Nested One-Way ANOVA, n = 5, $\alpha = 0.05$, ns: not significant;

p-values: * <= 0.1; ** <= 0.01; *** <=0.001; **** <=0.0001, each data point on the graph represents an image of the organoid along its z-axis)

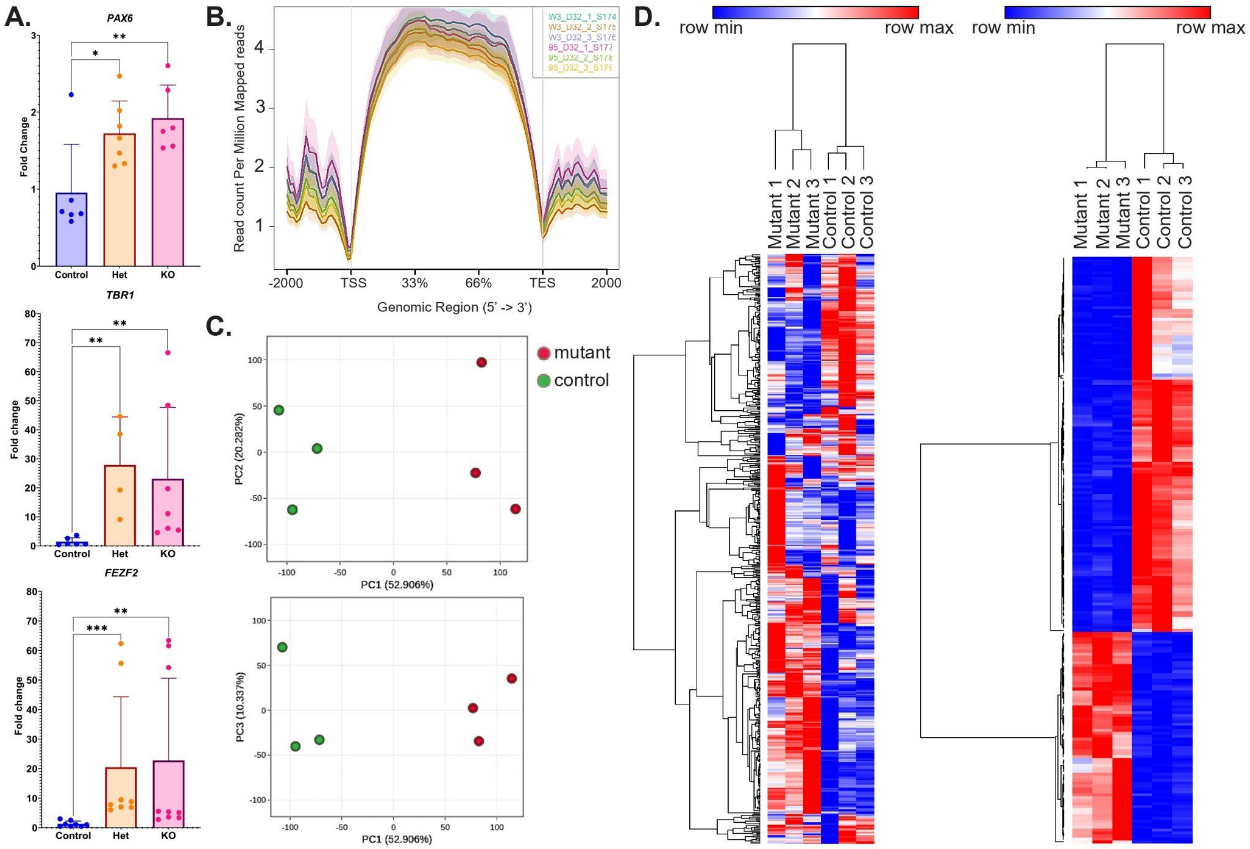

Supplementary Fig. 2

A. qPCR analysis derived from control, heterozygous, and mutant PRDM16 cortical organoids on day 32 revealed increased levels of *PAX6, TBR1* and *FEZF2* in heterozygous and homozygous mutants. **B.** Coverage plot of all RNA-seq samples. **C.** PCA plots of all RNA-seq samples. **D.** Heatmaps of all RNA-seq samples depict sample wise relationships of the Top 2000 expressed genes and top 200 DEGs identified by DESeq2. Each line represents a gene with normalized reads used as the input to generate this plot using Morpheus, https://software.broadinstitute.org/morpheus.

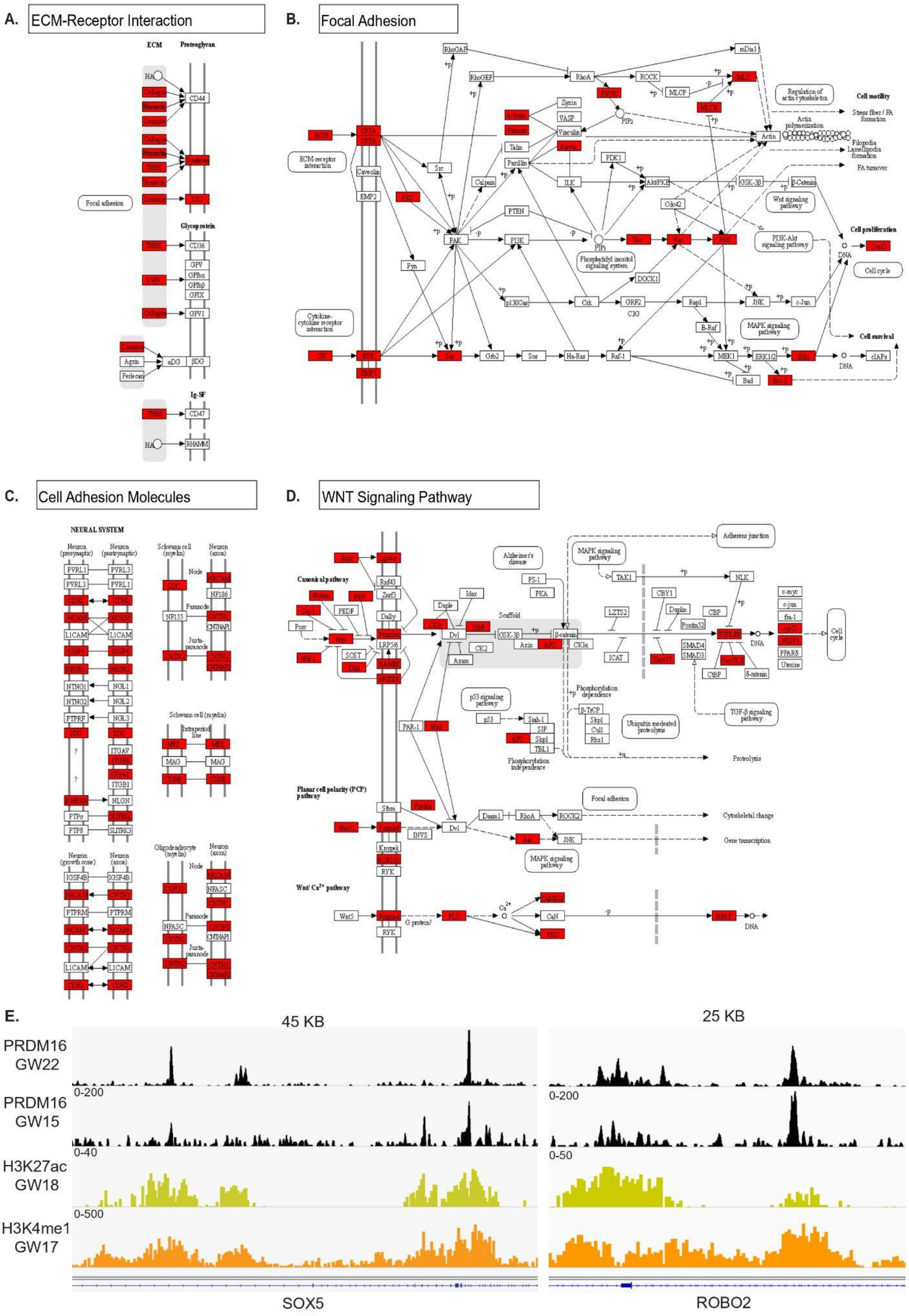
A.
ECM-Receptor Interaction
B.
Focal Adhesion
C.
Cell Adhesion Molecules
D.
WNT Signaling Pathway
E.
45 KB
25 KB
PRDM16
GW22
PRDM16
GW15
H3K27ac
GW18
H3K4me1
GW17
0-200
0-200
0-40
0-50
0-500
SOX5
ROBO2

Supplementary Fig. 3

A-D. KEGG plot view of all DEGs (red) identified in the mutant versus control organoid RNA-seq data shows multiple genes affected in all 4 exemplified pathways. **E.** Genomic tracks displaying the overlap of PRDM16 binding sites with enhancer marks H3K27ac and H3K4me1 within the *SOX5* and *ROBO2* loci in the human cortex

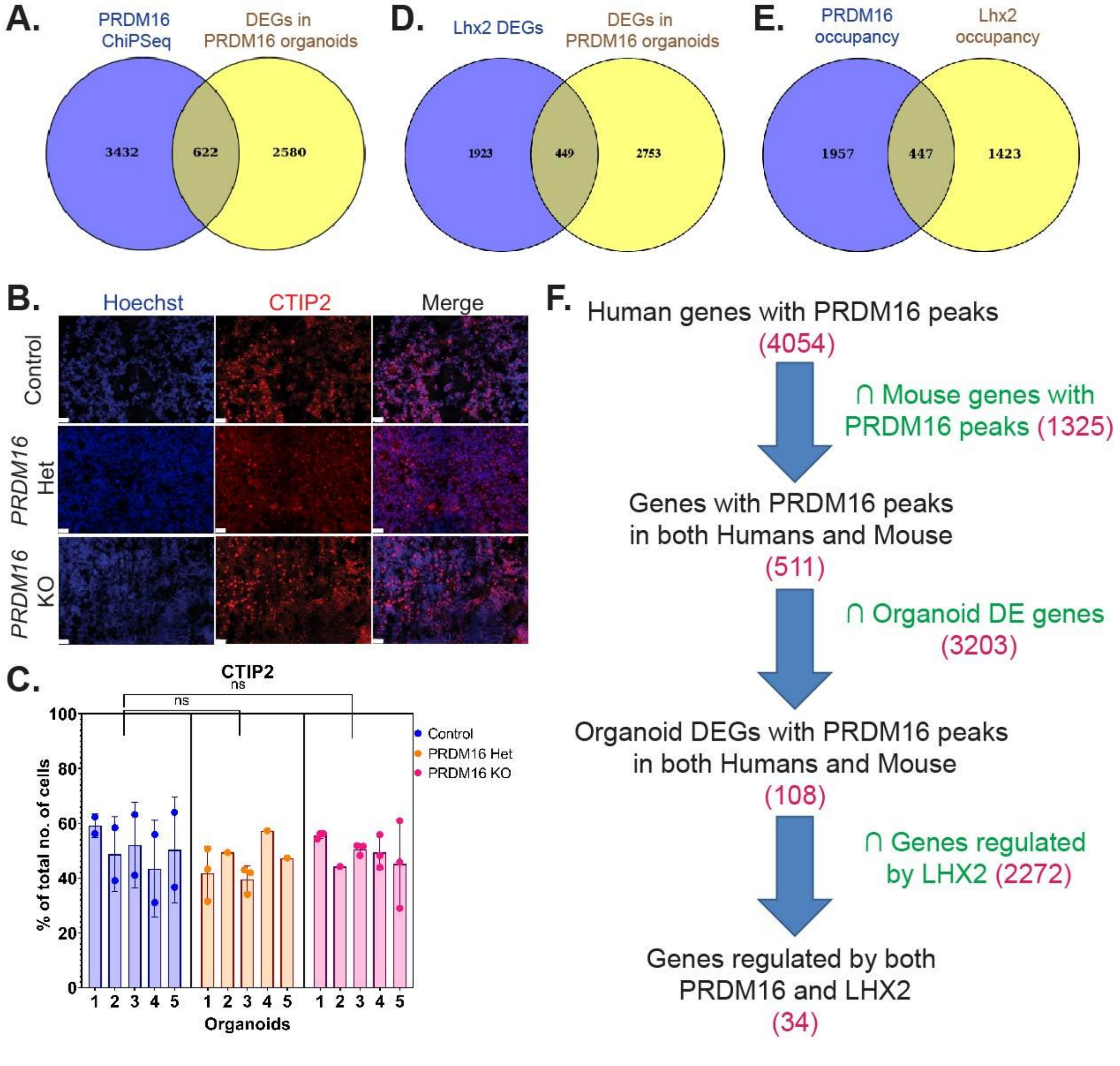

Supplementary Fig. 4

A. Venn diagram plot identifies genes that are occupied by PRDM16 in the ChIP-seq data and set of DEGs upon loss of PRDM16 in organoids **B.** Immunohistochemistry on 12 μm cryosections of day 32 control and PRDM16 mutant cortical organoids

showing the cortical neuronal markers CTIP2 (imaged using 25X objective lens, scale bar represents 20 μm) **C.** Percentage of the total number of cells that are CTIP2$^+$ neurons, in day 32 control and PRDM16 mutant cortical organoids (Nested One-Way ANOVA, n = 5, α = 0.05, each data point on the graph represents an image of the organoid along its z-axis) **D.** Venn diagram plot identifies dysregulated genes that are common upon loss of *Lhx2,* and loss of *PRDM16* in the organoids **E.** Venn diagram plot identifies a common set genes that are occupied by LHX2 and PRDM16 in the mouse. **F.** Sequential filtration of genes identifies a common gene regulatory network possibly regulated by both LHX2 and PRDM16.

Supplementary Fig. 5

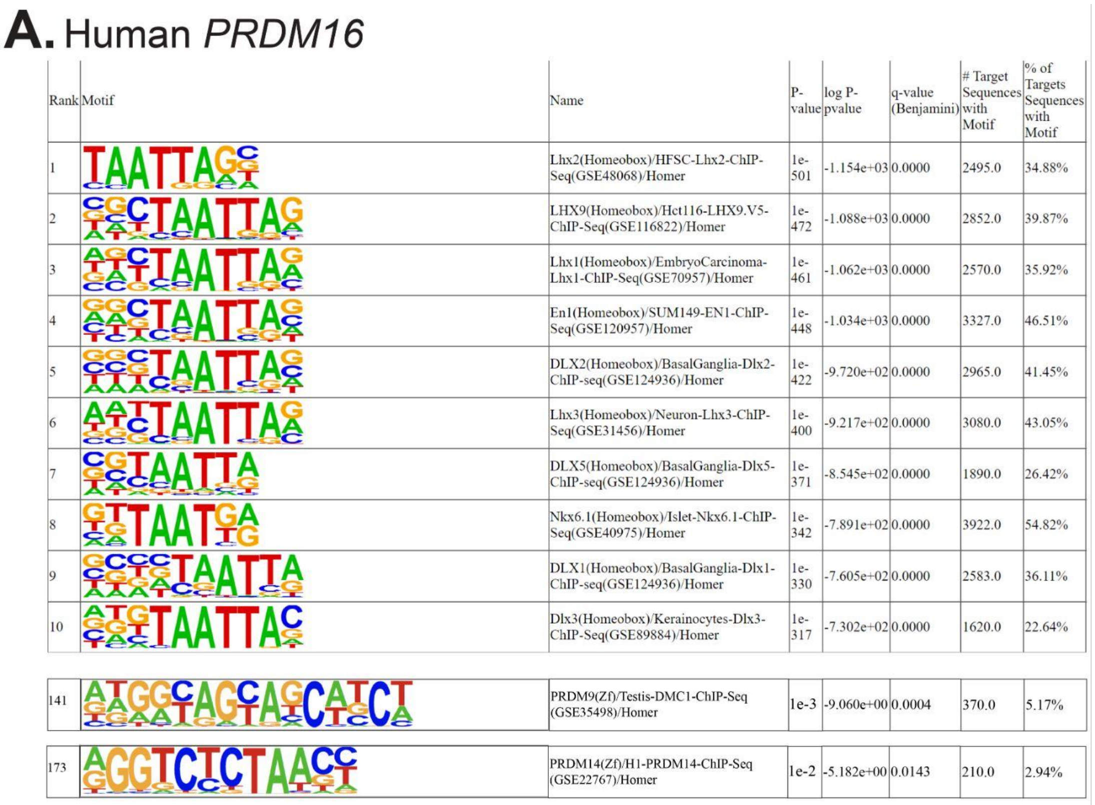

A. Human *PRDM16*

Rank	Motif	Name	P-value	log P-pvalue	q-value (Benjamini)	# Target Sequences with Motif	% of Targets Sequences with Motif
1		Lhx2(Homeobox)/HFSC-Lhx2-ChIP-Seq(GSE48068)/Homer	1e-501	-1.154e+03	0.0000	2495.0	34.88%
2		LHX9(Homeobox)/Hct116-LHX9.V5-ChIP-Seq(GSE116822)/Homer	1e-472	-1.088e+03	0.0000	2852.0	39.87%
3		Lhx1(Homeobox)/EmbryoCarcinoma-Lhx1-ChIP-Seq(GSE70957)/Homer	1e-461	-1.062e+03	0.0000	2570.0	35.92%
4		En1(Homeobox)/SUM149-EN1-ChIP-Seq(GSE120957)/Homer	1e-448	-1.034e+03	0.0000	3327.0	46.51%
5		DLX2(Homeobox)/BasalGanglia-Dlx2-ChIP-seq(GSE124936)/Homer	1e-422	-9.720e+02	0.0000	2965.0	41.45%
6		Lhx3(Homeobox)/Neuron-Lhx3-ChIP-Seq(GSE31456)/Homer	1e-400	-9.217e+02	0.0000	3080.0	43.05%
7		DLX5(Homeobox)/BasalGanglia-Dlx5-ChIP-seq(GSE124936)/Homer	1e-371	-8.545e+02	0.0000	1890.0	26.42%
8		Nkx6.1(Homeobox)/Islet-Nkx6.1-ChIP-Seq(GSE40975)/Homer	1e-342	-7.891e+02	0.0000	3922.0	54.82%
9		DLX1(Homeobox)/BasalGanglia-Dlx1-ChIP-seq(GSE124936)/Homer	1e-330	-7.605e+02	0.0000	2583.0	36.11%
10		Dlx3(Homeobox)/Kerainocytes-Dlx3-ChIP-Seq(GSE89884)/Homer	1e-317	-7.302e+02	0.0000	1620.0	22.64%
141		PRDM9(Zf)/Testis-DMC1-ChIP-Seq (GSE35498)/Homer	1e-3	-9.060e+00	0.0004	370.0	5.17%
173		PRDM14(Zf)/H1-PRDM14-ChIP-Seq (GSE22767)/Homer	1e-2	-5.182e+00	0.0143	210.0	2.94%

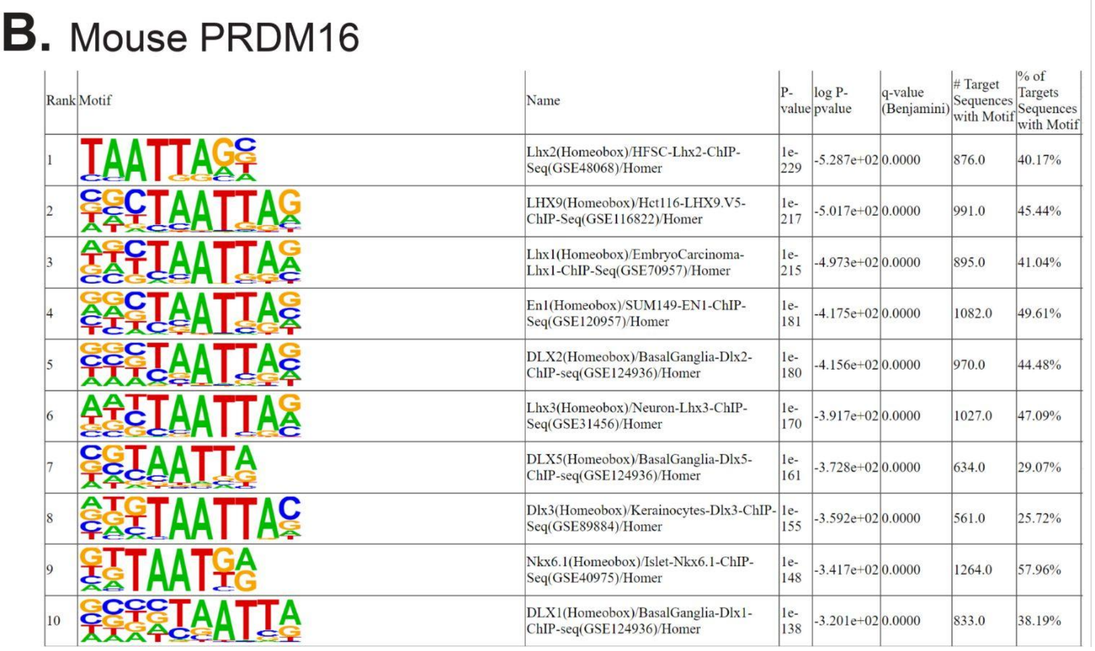

B. Mouse PRDM16

Rank	Motif	Name	P-value	log P-pvalue	q-value (Benjamini)	# Target Sequences with Motif	% of Targets Sequences with Motif
1		Lhx2(Homeobox)/HFSC-Lhx2-ChIP-Seq(GSE48068)/Homer	1e-229	-5.287e+02	0.0000	876.0	40.17%
2		LHX9(Homeobox)/Hct116-LHX9.V5-ChIP-Seq(GSE116822)/Homer	1e-217	-5.017e+02	0.0000	991.0	45.44%
3		Lhx1(Homeobox)/EmbryoCarcinoma-Lhx1-ChIP-Seq(GSE70957)/Homer	1e-215	-4.973e+02	0.0000	895.0	41.04%
4		En1(Homeobox)/SUM149-EN1-ChIP-Seq(GSE120957)/Homer	1e-181	-4.175e+02	0.0000	1082.0	49.61%
5		DLX2(Homeobox)/BasalGanglia-Dlx2-ChIP-seq(GSE124936)/Homer	1e-180	-4.156e+02	0.0000	970.0	44.48%
6		Lhx3(Homeobox)/Neuron-Lhx3-ChIP-Seq(GSE31456)/Homer	1e-170	-3.917e+02	0.0000	1027.0	47.09%
7		DLX5(Homeobox)/BasalGanglia-Dlx5-ChIP-seq(GSE124936)/Homer	1e-161	-3.728e+02	0.0000	634.0	29.07%
8		Dlx3(Homeobox)/Kerainocytes-Dlx3-ChIP-Seq(GSE89884)/Homer	1e-155	-3.592e+02	0.0000	561.0	25.72%
9		Nkx6.1(Homeobox)/Islet-Nkx6.1-ChIP-Seq(GSE40975)/Homer	1e-148	-3.417e+02	0.0000	1264.0	57.96%
10		DLX1(Homeobox)/BasalGanglia-Dlx1-ChIP-seq(GSE124936)/Homer	1e-138	-3.201e+02	0.0000	833.0	38.19%

A. Top 10 motifs and the two PRDM family motifs identified in the human PRDM16 ChIP-seq data **B.** Top 10 motifs identified in the mouse PRDM16 ChIP-seq data on regions that are conserved across the mouse and human peaks.
