# Supplementary material for: PRDM16 co-operates with LHX2 to shape the human brain
Source: Oxf Open Neurosci. 2024 Jan 24;3:kvae001. doi: 10.1093/oons/kvae001 (PMC10914218; doi:10.1093/oons/kvae001)
Supplement: Web_Material_kvae001 [file web_material_kvae001.zip › Reviewers comments - Supplementary Material - OXFNSC-2023-011.R2.docx]

**REVIEWER COMMENTS**

**OXFNSC-2023-011.R2 - PRDM16 co-operates with LHX2 to shape the human brain**

**Original submission - OXFNSC-2023-011**

**Reviewer 1**

Comments to the Author

In the manuscript entitled: “PRDM16 co-operates with LHX2 to shape the human brain” the authors aimed to model 1p36 deletion syndrome by scrutinizing the functional role of PRDM16 one of the key genes that are found in the 1p36 region. They perform a wide range of different experimental approaches from immunostainings to RNA sequencing and ChIP sequencing in human brain organoids and mice and they i) describe the patient phenotype upon introduction of de novo nonsense mutation in the PRDM16 coding sequence, ii) delve into the molecular mechanisms regulating cortical development via PRDM16 functional role. They show that PRDM16 mutant brain organoids exhibited altered cell cycle dynamics that affect mainly aRGs and the collaborative function of PRDM16 and LHX2 in regulating genes and biological pathways in neural progenitors.

This work is quite novel and interesting since it can contribute to the limited knowledge we still have on the molecular mechanisms that regulate early human corticogenesis and disease manifestation. The manuscript is well-written, and the work has been performed in a thorough and well-designed way. However, there are major considerations regarding the in-depth phenotype investigation and the patients' phenotype recapitulation in brain organoids and also on the focus of the authors mainly on aRGs and not the rest of the progenitors which have been also largely associated with the development of lissencephaly and microcephaly in humans (eg bRGs). I have some suggestions that I believe would be a useful improvement to the story.

Major comments:

- The authors mention that PRDM16 is co-expressed with both Pax6 and EOMES in the human developing cortex. Is then PRDM16 expressed in all neural progenitors? Which is the percentage of PRDM16+ cells expressing Pax6 and the one of EOMES? In different layers? In SVZ are there triple positive cells (PRDM16+Pax6+EOMES+)? Is PRDM16 characterized mainly aRGs, bRGs or IPs?

- In Fig.3 the images selected for presentation do not include nicely formed neuroepithelial structures. Is this typical in the mutant lines? If not, please show more representative pictures and lower magnification (this is also required in the control condition). Besides, the differences that the authors show are restricted to a specific layer in the developing brain organoids or not? Eg. VZ vs SVZ (apical vs basal progenitors). Bining analysis is recommended when doing such analysis. For instance, the authors could divide the cortical area into 3 bins (one to be closely correlated to VZ-, one to SVZ- and one to CP-like structures in organoids) and do the statistical analysis separately in the different areas. This should be also performed in the cell type markers the authors show in Fig2 given that none of the markers chosen (Sox2, Pax6, HopX) are specific for aRGs or bRGs, especially in such young stages organoids. This would be important if one would like to correlate the phenotype with a particular neural progenitor population and correlate it with the expression data shown in Fig.1.

- What about neurons? The authors do not investigate this thoroughly. Even though they do some analysis for Map2 and Tbr1 (Fig.5) this is not enough. Are there differences between deep vs upper layer neurons? What happens in older organoids when more neurons are generated? This is very important also because the authors mention cell cycle exit. Do they suggest that they show premature differentiation which eventually leads to progenitor exhaustion? This could help in understanding how the PRDM16 mutant organoids can recapitulate microcephaly and lissencephaly that the patients have. The authors need to perform additional experiments (in additional stages) in that direction to validate their model and scrutinize the phenotype in depth.

- Regarding the molecular mechanisms, the authors managed to scale down to 34 the most relevant candidate genes via which PRDM16 and Lhx2 may function to regulate cortical development. Have they thought of validating if some of them are key candidate genes? Possible rescue experiments could help in answering this question. Besides, are there any human-specific targets (eg. Genes that are human-specific or exhibit human-specific expression in the brain)? Although the authors discuss the similarities and possibly the differences between mouse and human mechanisms, they do not present robust data towards one or the other. It would be important to find potential targets that would explain the human patient phenotype (microcephaly-lissencephaly) and to recapitulate this or correlate this with mice.

- One key question is regarding bRGs. The authors choose not the investigate this at all even though this is the population that is mostly affected in mutant organoids (more than 50% reduction vs smaller increase in the aRGs – Fig.2) and is correlated with the expansion of the human cortex and folding. One could think of checking in the sequencing datasets in order to depict potential targets that are differentially regulated and are associated with bRGs. It is not clear to me why the authors only briefly discuss this population and focus mainly on aRGs. If the phenotype is not correlated with bRGs the authors should at least show it with some even short analysis. If, however this is not the case, then I believe that the authors miss one very important part of the phenotype/mechanism that would be essential for the story.

- Finally, what about delamination/apical belt integrity? The authors show quantifications of markers like ctnnb1, n-cad and also “cell adhesion” is amongst the highest biological processes affected in the PRDM16 mutant organoids. This together with the fact that they show more aRGs and less bRG could indicate a potential delamination problem. The authors should consider investigating more this angle and discuss it in the manuscript.

Minor comments:

- In Fig.1C the expression of Paax6 is depicted, not the one of Hes1 as mentioned in the text (page 4 line 2). Please correct accordingly.

- In Fig.2B there is a typo in the legend. In Fig2B only Pax6+ cells are depicted in the graph not Sox2. Please check and correct accordingly.

**Reviewer 2**

Comments to the Author

In their study, the authors addressed an important question in neural development and disease. What is the role of PRDM16 in human brain development? The authors first analyzed PRDM16 expression in human brain and found substantial expression levels in apical progenitor cells (i.e. apical radial glial cells). The authors also had access to clinical data and identified mutations in PRDM16 that seemed relevant. Next, the authors devised cerebral organoids from hESCs and introduced heterozygous and homozygous mutations to compare to respective controls. Interestingly, they found an increase in proliferating cells in PRDM16 KO organoids. Next the authors profiled gene expression and identified components of the WNT pathway and cell adhesion to be deregulated. The authors also carried out PRDM16 ChIP-seq whereby they compared human to mouse data. Lastly, the authors identified LHX2 as an interacting protein and propose that PRDM16 in concert with LHX2 regulate important steps in human neurogenesis and development.

This manuscript is excellent! The data is very convincing and neatly presented. I do not see any critical issues that require attention and recommend the manuscript for publication as is.

**Decision letter - OXFNSC-2023-011**

28-Sep-2023

Dear Dr. Reiner,

Manuscript ID OXFNSC-2023-011 entitled "PRDM16 co-operates with LHX2 to shape the human brain" which you submitted to the Oxford Open Neuroscience, has been reviewed. The comments of the reviewer(s) are included at the bottom of this letter.

The reviewers have recommended publication, but also suggest some revisions to your manuscript. Therefore, I invite you to respond to the reviewers' comments and revise your manuscript within the next 30 days of todays date.

Please note that this journal operates with transparent peer review. This means that if your submission is accepted for publication, the full peer review history of your article will publish online alongside your article. This includes reviewer comments, editor decision letters, and your author responses.

To revise your manuscript, log into https://mc.manuscriptcentral.com/oxfnsc and enter your Author Centre, where you will find your manuscript title listed under "Manuscripts with Decisions." Under "Actions," click on "Create a Revision." Your manuscript number has been appended to denote a revision.

You may also click the below link to start the revision process (or continue the process if you have already started your revision) for your manuscript. If you use the below link you will not be required to login to ScholarOne Manuscripts.

*** PLEASE NOTE: This is a two-step process. After clicking on the link, you will be directed to a webpage to confirm. ***

https://mc.manuscriptcentral.com/oxfnsc?URL_MASK=f990851c84294ea69474995a65864036

You will be unable to make your revisions on the originally submitted version of the manuscript. Instead, revise your manuscript using a word processing program and save it on your computer. Please also highlight the changes to your manuscript within the document by using the track changes mode in MS Word or by using bold or colored text.

Once the revised manuscript is prepared, you can upload it and submit it through your Author Centre.

When submitting your revised manuscript, you will be able to respond to the comments made by the reviewer(s) in the space provided. You can use this space to document any changes you make to the original manuscript. In order to expedite the processing of the revised manuscript, please be as specific as possible in your response to the reviewer(s).

IMPORTANT: Your original files are available to you when you upload your revised manuscript. Please delete any redundant files before completing the submission.

Because we are trying to facilitate timely publication of manuscripts submitted to the Oxford Open Neuroscience, your revised manuscript should be uploaded within the next 30 days from todays date. If it is not possible for you to submit your revision in a reasonable amount of time, we may have to consider your paper as a new submission.

Once again, thank you for submitting your manuscript to the Oxford Open Neuroscience and I look forward to receiving your revision.

Sincerely,

Dr. Alicia Izquierdo

Senior Editor, Oxford Open Neuroscience

aizquie@psych.ucla.edu

Reviewer: 1

Comments to the Author

In the manuscript entitled: “PRDM16 co-operates with LHX2 to shape the human brain” the authors aimed to model 1p36 deletion syndrome by scrutinizing the functional role of PRDM16 one of the key genes that are found in the 1p36 region. They perform a wide range of different experimental approaches from immunostainings to RNA sequencing and ChIP sequencing in human brain organoids and mice and they i) describe the patient phenotype upon introduction of de novo nonsense mutation in the PRDM16 coding sequence, ii) delve into the molecular mechanisms regulating cortical development via PRDM16 functional role. They show that PRDM16 mutant brain organoids exhibited altered cell cycle dynamics that affect mainly aRGs and the collaborative function of PRDM16 and LHX2 in regulating genes and biological pathways in neural progenitors.

This work is quite novel and interesting since it can contribute to the limited knowledge we still have on the molecular mechanisms that regulate early human corticogenesis and disease manifestation. The manuscript is well-written, and the work has been performed in a thorough and well-designed way. However, there are major considerations regarding the in-depth phenotype investigation and the patients' phenotype recapitulation in brain organoids and also on the focus of the authors mainly on aRGs and not the rest of the progenitors which have been also largely associated with the development of lissencephaly and microcephaly in humans (eg bRGs). I have some suggestions that I believe would be a useful improvement to the story.

Major comments:

- The authors mention that PRDM16 is co-expressed with both Pax6 and EOMES in the human developing cortex. Is then PRDM16 expressed in all neural progenitors? Which is the percentage of PRDM16+ cells expressing Pax6 and the one of EOMES? In different layers? In SVZ are there triple positive cells (PRDM16+Pax6+EOMES+)? Is PRDM16 characterized mainly aRGs, bRGs or IPs?

- In Fig.3 the images selected for presentation do not include nicely formed neuroepithelial structures. Is this typical in the mutant lines? If not, please show more representative pictures and lower magnification (this is also required in the control condition). Besides, the differences that the authors show are restricted to a specific layer in the developing brain organoids or not? Eg. VZ vs SVZ (apical vs basal progenitors). Bining analysis is recommended when doing such analysis. For instance, the authors could divide the cortical area into 3 bins (one to be closely correlated to VZ-, one to SVZ- and one to CP-like structures in organoids) and do the statistical analysis separately in the different areas. This should be also performed in the cell type markers the authors show in Fig2 given that none of the markers chosen (Sox2, Pax6, HopX) are specific for aRGs or bRGs, especially in such young stages organoids. This would be important if one would like to correlate the phenotype with a particular neural progenitor population and correlate it with the expression data shown in Fig.1.

- What about neurons? The authors do not investigate this thoroughly. Even though they do some analysis for Map2 and Tbr1 (Fig.5) this is not enough. Are there differences between deep vs upper layer neurons? What happens in older organoids when more neurons are generated? This is very important also because the authors mention cell cycle exit. Do they suggest that they show premature differentiation which eventually leads to progenitor exhaustion? This could help in understanding how the PRDM16 mutant organoids can recapitulate microcephaly and lissencephaly that the patients have. The authors need to perform additional experiments (in additional stages) in that direction to validate their model and scrutinize the phenotype in depth.

- Regarding the molecular mechanisms, the authors managed to scale down to 34 the most relevant candidate genes via which PRDM16 and Lhx2 may function to regulate cortical development. Have they thought of validating if some of them are key candidate genes? Possible rescue experiments could help in answering this question. Besides, are there any human-specific targets (eg. Genes that are human-specific or exhibit human-specific expression in the brain)? Although the authors discuss the similarities and possibly the differences between mouse and human mechanisms, they do not present robust data towards one or the other. It would be important to find potential targets that would explain the human patient phenotype (microcephaly-lissencephaly) and to recapitulate this or correlate this with mice.

- One key question is regarding bRGs. The authors choose not the investigate this at all even though this is the population that is mostly affected in mutant organoids (more than 50% reduction vs smaller increase in the aRGs – Fig.2) and is correlated with the expansion of the human cortex and folding. One could think of checking in the sequencing datasets in order to depict potential targets that are differentially regulated and are associated with bRGs. It is not clear to me why the authors only briefly discuss this population and focus mainly on aRGs. If the phenotype is not correlated with bRGs the authors should at least show it with some even short analysis. If, however this is not the case, then I believe that the authors miss one very important part of the phenotype/mechanism that would be essential for the story.

- Finally, what about delamination/apical belt integrity? The authors show quantifications of markers like ctnnb1, n-cad and also “cell adhesion” is amongst the highest biological processes affected in the PRDM16 mutant organoids. This together with the fact that they show more aRGs and less bRG could indicate a potential delamination problem. The authors should consider investigating more this angle and discuss it in the manuscript.

Minor comments:

- In Fig.1C the expression of Paax6 is depicted, not the one of Hes1 as mentioned in the text (page 4 line 2). Please correct accordingly.

- In Fig.2B there is a typo in the legend. In Fig2B only Pax6+ cells are depicted in the graph not Sox2. Please check and correct accordingly.

Reviewer: 2

Comments to the Author

In their study, the authors addressed an important question in neural development and disease. What is the role of PRDM16 in human brain development? The authors first analyzed PRDM16 expression in human brain and found substantial expression levels in apical progenitor cells (i.e. apical radial glial cells). The authors also had access to clinical data and identified mutations in PRDM16 that seemed relevant. Next, the authors devised cerebral organoids from hESCs and introduced heterozygous and homozygous mutations to compare to respective controls. Interestingly, they found an increase in proliferating cells in PRDM16 KO organoids. Next the authors profiled gene expression and identified components of the WNT pathway and cell adhesion to be deregulated. The authors also carried out PRDM16 ChIP-seq whereby they compared human to mouse data. Lastly, the authors identified LHX2 as an interacting protein and propose that PRDM16 in concert with LHX2 regulate important steps in human neurogenesis and development.

This manuscript is excellent! The data is very convincing and neatly presented. I do not see any critical issues that require attention and recommend the manuscript for publication as is.

**Author response**

The response letter is an attached file.

**Revised Submission - OXFNSC-2023-011.R1**

**Decision letter - OXFNSC-2023-011.R1**

11-Nov-2023

Dear Dr. Reiner,

Manuscript ID OXFNSC-2023-011.R1 entitled "PRDM16 co-operates with LHX2 to shape the human brain" which you submitted to the Oxford Open Neuroscience, has been reviewed. The comments of the reviewer(s) are included at the bottom of this letter.

Please note that this journal operates with transparent peer review. This means that if your submission is accepted for publication, the full peer review history of your article will publish online alongside your article. This includes reviewer comments, editor decision letters, and your author responses.

The reviewer(s) have recommended publication, but also suggest some minor revisions to your manuscript. Therefore, I invite you to respond to the reviewer(s)' comments and revise your manuscript within the next 30 days from todays date.

To revise your manuscript, log into https://mc.manuscriptcentral.com/oxfnsc and enter your Author Centre, where you will find your manuscript title listed under "Manuscripts with Decisions." Under "Actions," click on "Create a Revision." Your manuscript number has been appended to denote a revision.

You may also click the below link to start the revision process (or continue the process if you have already started your revision) for your manuscript. If you use the below link you will not be required to login to ScholarOne Manuscripts.

*** PLEASE NOTE: This is a two-step process. After clicking on the link, you will be directed to a webpage to confirm. ***

https://mc.manuscriptcentral.com/oxfnsc?URL_MASK=ac263abcc5bf4ec38b4c1b8565a7e2b9

You will be unable to make your revisions on the originally submitted version of the manuscript. Instead, revise your manuscript using a word processing program and save it on your computer. Please also highlight the changes to your manuscript within the document by using the track changes mode in MS Word or by using bold or colored text.

Once the revised manuscript is prepared, you can upload it and submit it through your Author Centre.

When submitting your revised manuscript, you will be able to respond to the comments made by the reviewer(s) in the space provided. You can use this space to document any changes you make to the original manuscript. In order to expedite the processing of the revised manuscript, please be as specific as possible in your response to the reviewer(s).

IMPORTANT: Your original files are available to you when you upload your revised manuscript. Please delete any redundant files before completing the submission.

Because we are trying to facilitate timely publication of manuscripts submitted to the Oxford Open Neuroscience, your revised manuscript should be uploaded within the next 30 days from todays date. If it is not possible for you to submit your revision in a reasonable amount of time, we may have to consider your paper as a new submission.

Once again, thank you for submitting your manuscript to the Oxford Open Neuroscience and I look forward to receiving your revision.

Sincerely,

Dr. Alicia Izquierdo

Senior Editor, Oxford Open Neuroscience

aizquie@psych.ucla.edu

Reviewer: 1

Comments to the Author

The authors of the manuscript titled “PRDM16 co-operates with LHX2 to shape the human brain”, although in their response to the editor wrote that they valued the points I raised in my review, in the revised draft they submitted, they opted not to include any new data, or address most of my points, claiming that this would require a significant extension of the project timeline and that it would be out of the scope of their study. Although I appreciate their view, I do not fully agree.

Specifically:

- they have addressed points 1 and 2.

- for point 3, they have not included more analysis on their comparison between control and mutant organoids using binning analysis (for markers included in Fig.2 and Fig 3). This is a typical way to analyse the phenotype observed which gives detailed information on the distribution of different cell types. Besides, it does not require more experiments/data but only a re-analysis of the existing ones. Therefore, I think that this point should be fully addressed for the submission to be published.

- The four points 4-7 were not addressed. I understand that addressing them may necessitate additional effort. However, I think that at least point 4, besides point 3 above, should be addressed, by including a comparison of the neuronal population between control and mutant organoids at one later time point.

Associate Editor

Comments to the Author:

Please see remaining critique from one of the Reviewers which necessitates re-analyses but not new experiments.

**Author response**

We thank the editors for providing us with the opportunity to improve our manuscript. We hope that the current changes are sufficient.

We want to thank the reviewer for taking the time to re-review our manuscript. We have taken into consideration the comments and have conducted re-analyses but not new experiments.

More specifically, we conducted a binned-type analysis and modified 2 main figures (fig 2 and fig 3) and 1 supplementary figure (Supp. fig 1), the associated modified text is highlighted in color.

**Revised Submission - OXFNSC-2023-011.R2**

**Decision letter - OXFNSC-2023-011.R2**

15-Dec-2023

Dear Dr. Reiner,

It is a pleasure to accept your revised manuscript entitled "PRDM16 co-operates with LHX2 to shape the human brain" in its current form for publication in the Oxford Open Neuroscience. The comments of the reviewer(s) who reviewed your manuscript are included at the foot of this letter.

Please note that this journal operates with transparent peer review. This means that the full peer review history of your article will publish online alongside your article. This includes reviewer comments, editor decision letters, and your author responses.

Next steps

You will receive an email from no-reply@scipris.com within roughly one week. This is your invitation to sign up for an account with SciPris, Oxford University Press’ author portal hosted by Aptara. You will need to create an account if you do not already hold one. Please register or log into your account and follow the online instructions which will guide you through signing your licence and paying the APC. The email and the portal have clearly signposted support options if you need any help during this process.

Please note that SciPris is a completely different system from ScholarOne, so your credentials to submit your manuscript here will not work there. Once you’ve created a SciPris account, you will be able to use it whenever you publish with Oxford Open Neuroscience or any OUP journal. Please note that OUP will only ever request payment for applicable fees be made via SciPris or to an OUP bank account. If you ever have concerns about the legitimacy of a request, please do not hesitate to contact a customer services agent via the SciPris portal or directly via oupsupport@scipris.com.

Thank you for your fine contribution. On behalf of the Editors of the Oxford Open Neuroscience, we look forward to your continued contributions to the Journal.

Sincerely,

Dr. Alicia Izquierdo

Senior Editor, Oxford Open Neuroscience

aizquie@psych.ucla.edu

Reviewer: 1

Comments to the Author

In the revised manuscript authors performed some of my suggestions. Although the authors decided not to do more experiments, overall, they addressed some of my main concerns. I do not have any other comments.

Associate Editor

Comments to the Author:

(There are no comments.)
